# YAP Levels Combined with Plasma CEA Levels Are Prognostic Biomarkers for Early-Clinical-Stage Patients of Colorectal Cancer

**DOI:** 10.1155/2019/2170830

**Published:** 2019-11-26

**Authors:** Zihui Xu, Hui Wang, Ling Gao, Hongfeng Zhang, Xiangyang Wang

**Affiliations:** ^1^Department of Endocrinology, Renmin Hospital of Wuhan University, Wuhan, China; ^2^Department of Gastrointestinal Surgery, The Central Hospital of Wuhan, Tongji Medical College, Huazhong University of Science and Technology, Wuhan, China; ^3^Department of Pathology, The Central Hospital of Wuhan, Tongji Medical College, Huazhong University of Science and Technology, Wuhan, China; ^4^Key Laboratory for Molecular Diagnosis of Hubei Province, The Central Hospital of Wuhan, Tongji Medical College, Huazhong University of Science and Technology, Wuhan, China

## Abstract

**Background:**

Colorectal cancer (CRC) is one of the most common cancers worldwide. Surgical operation is routinely applied to patients with CRC. An important part of postoperative care for patients is to assess the prognosis of patients, especially those with early-stage cancers. However, effective biomarkers for CRC prognosis remain inadequate. The purpose of this study was to assess the prognostic potential of Yes-associated protein (YAP) and carcinoembryonic antigen (CEA) in early-stage CRC.

**Methods:**

A total of 116 matched pairs of CRC tissues and adjacent normal mucosae as well as 73 cases of metastatic lymph nodes were analyzed.

**Results:**

The results show that CRC tissues exhibited higher YAP expression compared with the adjacent normal mucosae. Immunohistochemical analysis shows that YAP expression in the CRC or lymphatic metastatic tissues was clearly higher than that in normal mucosae (*P* < 0.01), whereas that in CRC tissues with lymphatic metastasis was higher than that in tissues without lymphatic metastasis (*P* < 0.05). YAP expression is associated with serosal invasion, lymphatic metastasis, lymph node ratio, remote metastasis, Dukes stage, and CEA levels (*P* < 0.05). YAP and CEA are independent predictors of the survival of CRC patients (*P* < 0.05 and *P* < 0.01). YAP predicted CRC prognosis primarily for patients with late-clinical-stage CRC (*P*=0.002), but not for patients with early-clinical-stage CRC (*P*=0.083). However, patients with high YAP and high CEA levels exhibited lower overall survival rates than those with low YAP expression in early-clinical-stage CRC (*P* < 0.001).

**Conclusion:**

High YAP levels in the cancer tissues combined with high plasma CEA levels are potential biomarkers for predicting CRC prognosis in the early clinical stage.

## 1. Introduction

Colorectal cancer (CRC) is the most common malignant tumor of the digestive system. CRC ranks fourth among malignant tumors [1]. Although significant progress has been made in the treatment of CRC, the incidence and mortality are still high due to the frequent recurrence and metastasis [2]. At present, early surgical resection is the best strategy for the treatment of resectable tumors. However, about 20% of CRC patients relapse within five years after surgery [3]. Early screening and detection of CRC is an important clinical strategy for improving long-term survival. In addition, effective biomarkers are also needed to detect early recurrence during follow-up. Therefore, it is urgent to search for effective biomarkers for early prognostic estimations of CRC [4].

The levels of tumor biomarkers in cancer tissues and plasma can reflect the progression and prognosis of malignant tumor. Yes-associated protein (YAP) is a downstream effector molecule of emerging tumor suppressor pathway called Hippo [5]. An increasing number of studies suggest that YAP is an oncogenic transcription coactivator that is highly expressed in various tumors, which regulates tumor development and progression [6]. Patients with higher YAP expression showed a trend of shorter survival times [7]. Another tumor biomarker, carcinoembryonic antigen (CEA), is an indicator of metastasis in a broad spectrum of neoplastic diseases [8]. It is mainly used for assistant diagnosis of malignant tumors, determining prognosis, and monitoring curative effect and recurrence of tumors [9]. However, its specificity is poor and its role in early diagnosis is not obvious. It may improve the positive rate of cancer diagnosis by combining with other biomarkers. In this study, we investigated the YAP expression in CRC tissues and plasma CEA levels, as well as their correlation with CRC progression and prognosis.

## 2. Materials and Methods

### 2.1. Patients and Specimens

A total of 116 cases of paraffin-embedded CRC specimens were collected from January 2010 to May 2012. These specimens were all from patients diagnosed in The Central Hospital of Wuhan. All patients were not subjected to chemotherapy or radiotherapy prior to surgery. Complete clinical data were obtained during a five-year follow-up period. The clinicopathologic features are summarized in Table 1. The mean age of the patients was 61 years (range, 21 to 83 years), and the mean tumor size was 6.1 cm (range, 1.2 cm to 30 cm). 60 (51%) of the patients died within the follow-up period, whereas 26 (22.4%) suffered from distant metastasis. The fresh clinical samples consisting of tumor tissues and adjacent normal mucosae were obtained; the latter was resected at least 1 cm from the edge of the tumor. These samples were processed immediately after the surgical operation and were then stored in liquid nitrogen. These samples were acquired with patient consent as well as approval from the Ethics Committee of The Central Hospital of Wuhan.

### 2.2. RNA Isolation and Quantitative Real-Time Polymerase Chain Reaction (qPCR)

Total RNA was extracted from frozen tissues using Trizol reagent (Invitrogen, Carlsbad, CA, USA) according to the manufacturer's instructions. Reverse transcription was performed using the high-capacity cDNA RT kit (Applied Biosystems, CA, USA) on an access RT system (Promega, WI, USA) according to the manufacturer's protocol. qPCR was performed using a SYBR Green Master Mix kit (Applied Biosystems, CA, USA) according to the manufacturer's instructions. All experiments were performed in triplicate. *β*-Actin was used as the internal control. Data were analyzed using the comparative Ct method (2^−ΔΔCt^). The primer sequences were as follows: *β*-actin forward: 5ʹ-ATAGCACAGCCTGGATAGCAACGTAC-3ʹ, reverse: 5ʹ-CACCTTCTACAATGAGCTGCGTGTG-3ʹ; and YAP forward: 5ʹ-CGCTCTTCAACGCCGTCA-3ʹ, reverse: 5ʹ-AGTACTGGCCTGTCGGGAGT-3ʹ.

### 2.3. Western Blot Analysis

CRC and normal mucosa tissues, which were stored in liquid nitrogen, were ground into powder, placed into electrolytically polished tubes, and were then lysed on ice in a radioimmunoprecipitation assay buffer (Pierce, Rockford, IL, USA) with protease inhibitors (Pierce, Rockford, IL, USA). The protein concentration was quantified via the bicinchoninic acid method. The proteins were placed into different lanes, subjected to electrophoresis, electrotransferred to polyvinylidene difluoride membranes (Millipore, Bedford, MA, USA), and then blocked with 5% nonfat dry milk. The membranes were incubated overnight with anti-YAP polyclonal antibodies (1 : 1000 dilution; Santa Cruz Biotechnology, Santa Cruz, CA, USA) at 4°C and then incubated with horseradish peroxidase (HRP)-conjugated secondary antibodies (1 : 5000 dilution; Invitrogen, Carlsbad, CA, USA). The signals were visualized via enhanced chemiluminescence (Pierce, Rockford, IL, USA). *β*-Actin was used as the internal control. The total densitometric quantification of the bands was measured using the AlphaEaseFC software tool (Alpha Innotech, San Leandro, CA, USA).

### 2.4. Immunohistochemical Analysis

Paraffin sections of 4 *μ*m thickness were baked at 68°C for at least 30 min. The sections were then deparaffinized and hydrated in graded xylene and ethanol, respectively, prior to immunostaining. During hydration, endogenous peroxidase was blocked in 0.3% H_2_O_2_ diluted with double-distilled water for 15 min. The paraffin sections (4 *μ*m thick) were then heated in a citrate buffer (pH 6.0) for 15 min at 100°C using a microwave oven as the heat source. The paraffin sections were naturally cooled in the citrate buffer, rinsed in double-distilled water five times, and then immersed in Tris-buffered saline (pH 7.5) for 5 min. A blocking solution (bovine serum albumin diluted with Tris-buffered saline, pH 7.5) was used to block the paraffin sections at room temperature for 30 min. The sections were incubated overnight with primary anti-YAP polyclonal antibodies (1 : 200 dilution; Santa Cruz Biotechnology, Santa Cruz, CA, USA) at 4°C. HRP-conjugated antibodies against rabbit IgG (1 : 1000 dilution; Invitrogen, Carlsbad, CA, USA) were then added, and the sections were incubated at 4°C for 40 min. The sections were developed in a diaminobenzidine solution for 5 min and then counterstained in hematoxylin for 5 min. Finally, the sections were dehydrated in graded ethanol and xylene. The stained sections were separately scored by two pathologists according to the scoring method used in previous studies [10]. The staining extents were graded as 0 (0%), 1 (≤25%), 2 (26% to 50%), 3 (51% to 75%), and 4 (≥76%) according to the proportion of the positively stained area in the entire carcinoma-involved area or the entire section of the normal specimens. The staining intensities were graded as 0 (negative), 1 (weak), 2 (medium), and 3 (strong). The sum of the staining extent and intensity scores was obtained and then classified into four final levels of YAP expression: (−) indicates a final staining score of <3; (+), a final staining score of 3; (++), a final staining score of 4; and (+++), a final staining score of ≥5. Tumors with a final staining score of ≥3 were considered positive sections, whereas those with final staining scores of 0 to (+) were graded as sections with low YAP expression. Tumors with a final staining score of (++) to (+++) were graded as sections with high YAP expression.

### 2.5. Statistical Analysis

YAP expression levels in the CRC tissues, adjacent normal mucosae, and lymphatic metastatic tissues were analyzed using the paired-sample or two-sample *t*-test. The YAP protein levels and mRNA levels in the tumor and adjacent normal mucosae were compared using the paired-sample *t*-test. The correlation between YAP expression levels and the clinicopathologic features of CRC patients was analyzed using Pearson's chi-square test. The Cox proportional-hazards model in the multivariate analysis was used to analyze the correlation between the variables and survival. The overall survival time was calculated from the date of surgical operation to the date of death or the last follow-up date. The survival analyses were then performed according to the Kaplan-Meier plots and the log-rank test. All above-mentioned data were analyzed using the SPSS 17.0 software, where *P* < 0.05 is considered significant.

## 3. Results

### 3.1. YAP Level in the CRC Tissues and Adjacent Normal Mucosae

**Figure 1:**
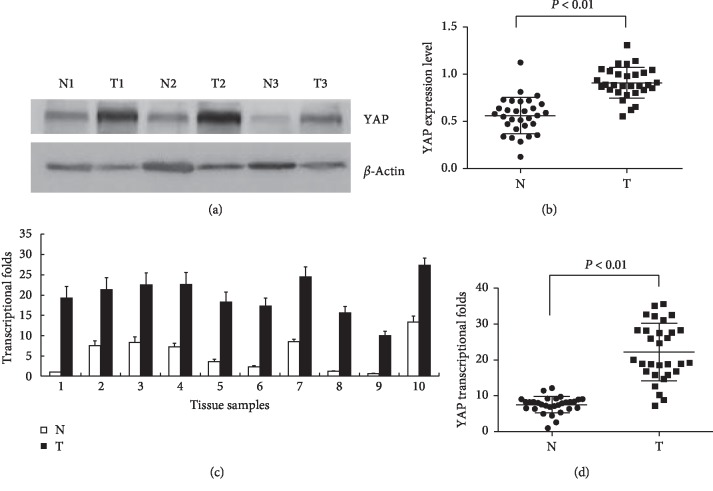
YAP expression in fresh colorectal cancer specimens. (a) Representative western blot analyses of YAP expression in tumor tissues T and the adjacent normal mucosae N. *β*-Actin was used as the control. (b) The relative YAP protein levels in 30 matched pairs of CRC tissues and the adjacent normal mucosae were calculated using the CRC tissue of sample 1 as the reference. Data are expressed as mean ± standard deviation (SD). The mean YAP protein level in the tumor tissues was significantly increased (*P* < 0.01) compared with that in the adjacent normal mucosae. (c) Representative quantitative real-time polymerase chain reaction (qPCR) of YAP mRNA expression in tumor tissues and the adjacent normal mucosae. *β*-Actin was used as the control. (d) The relative YAP mRNA levels in 30 matched pairs of CRC tissues and the adjacent normal mucosae were calculated using the adjacent normal mucosa of sample 1 as the reference. Data are expressed as mean ± SD. The mean fold changes of YAP in the tumors clearly increased compared with those in the adjacent normal mucosae (*P* < 0.01).

Western blot and qPCR were both used to detect whether YAP was highly expressed in the fresh clinical specimens of human CRC, including 30 matched pairs of CRC tissues and the adjacent normal mucosae. The YAP protein levels in 21 CRC tissues were upregulated compared with those in the adjacent normal mucosae. Only four or five CRC tissues exhibited nearly identical or low YAP expression compared with the matching adjacent normal mucosae, as indicated by western blotting results (Figures 1(a) and 1(b)). The YAP mRNA levels in 22 CRC tissues were upregulated compared with the matching adjacent normal mucosae, and only 3 or 5 CRC tissues showed nearly identical or low expression of YAP, as indicated by qPCR assay results (Figures 1(c) and 1(d)). The YAP protein and mRNA levels in tumor tissues were approximately 2 to 4 times higher than those in the matching adjacent normal mucosae (*P* < 0.01; *P* < 0.01).

### 3.2. Immunohistochemical (IHC) Analysis of YAP in Human CRC Specimens

**Figure 2:**
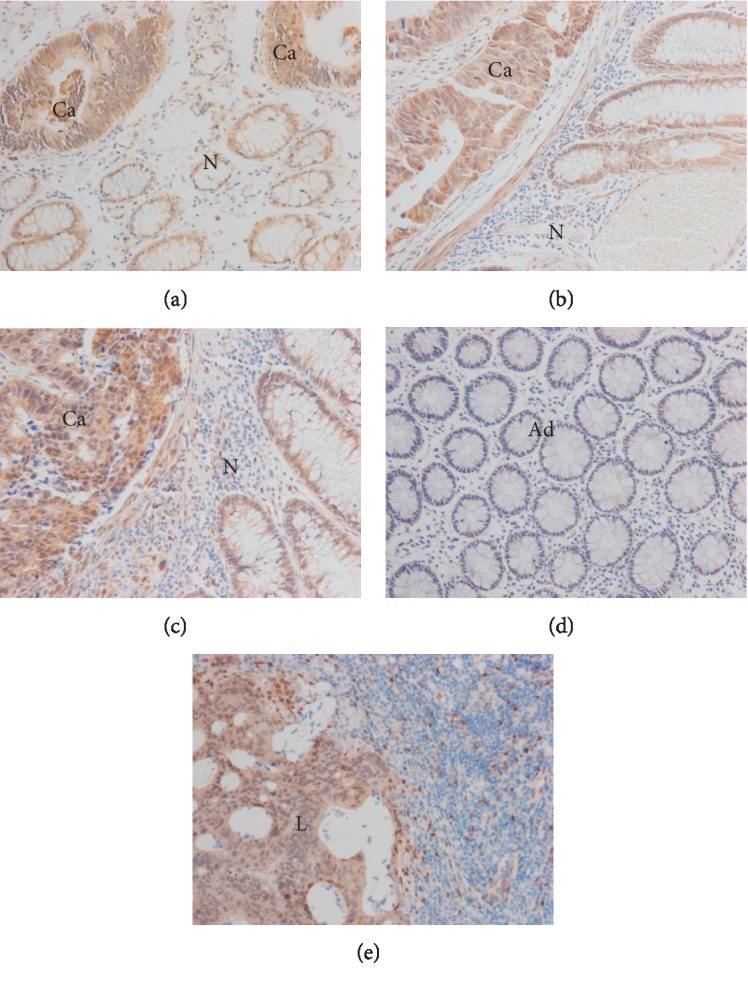
Immunohistochemical staining of YAP in CRC tissues, adjacent normal mucosae, and the corresponding lymphatic metastatic tissues. (a–c) Staining in the cancerous (Ca) and normal N parts in 3 representative sections (×200). (a) The staining extent and intensity scores were obtained and classified into final levels of YAP expression: (a, +), (b, ++), and (c, +++). (d) Negative staining of YAP in the adjacent normal mucosae (Ad) (×200). (e) Strong staining of YAP in the metastatic lymphatic gland (L) (×200).

IHC analysis was used to assess the YAP expression level in 116 matched pair of paraffin-embedded CRC specimens as well as in adjacent normal mucosae and 73 cases of metastatic lymph nodes to determine its clinical significance. YAP was mainly present in the CRC cell nuclei, with small amounts found in the cytoplasm. The high YAP expression in CRC tissues and low expression in normal mucosae were distinctly observed (Figures 2(a)–2(c)). By contrast, YAP was barely detected in the matching adjacent normal mucosae. Weak or even negative signals for YAP were mainly observed in adjacent normal mucosae (Figure 2(d)), whereas positive staining signals were detected in the lymphatic metastatic tissues (Figure 2(e)). In 116 CRC specimens, 76 cases (65.5%) exhibited high YAP expression (YAP 2+ to 3+), whereas the other 40 cases (34.5%) exhibited low expression (YAP 0 to 1+). The correlation between YAP expression in CRC tissues and clinicopathologic features is as follows: YAP expression level was closely related to serosal invasion (*P*=0.040), lymphatic metastasis (*P*=0.013), the lymph node ratio (LNR; *P*=0.035), remote metastasis (*P*=0.020), Dukes stage (*P*=0.033), and the CEA (*P*=0.011) (Table 1). Conversely, YAP expression level showed no correlation with age (*P*=0.706), gender (*P*=0.067), tumor size (*P*=0.543), and differentiation (*P*=0.712) (Table 1). YAP expression in CRC or lymphatic metastatic tissues was clearly higher than that in the adjacent normal mucosae (*Z* = 16.037, *P* < 0.001; *Z* = 9.322, *P* < 0.001; Table 2). YAP expression levels in the CRC tissues with lymphatic metastasis and lymphatic metastatic tissues were nearly identical (*Z* = −2.034 *P* = 0.094; Table 2). The expression levels in the CRC tissues with lymphatic metastasis were clearly higher than those without lymphatic metastasis (*Z* = 4.875, *P*=0.017; Table 2). These data show that YAP high expression may be related to CRC progression.

### 3.3. Relationship between YAP, CEA, and Overall Survival of CRC Patients

The Cox proportional-hazards model was built to determine whether YAP and CEA are independent prognostic factors for the overall survival of CRC patients. Based on the single variable analysis, the relevant prognostic factors were the high YAP expression level (*P*=0.001), Dukes stage (C and D) (*P* < 0.001), remote metastasis (*P*=0.001), and CEA (>5 ng/ml) (*P*=0.029) (Table 3). Multivariate analysis results indicate that high YAP expression, Dukes stage (C and D), and remote metastasis, but not CEA (>5 ng/ml), play independent prognostic roles in predicting the overall survival of patients with CRC (*P*=0.026, *P*=0.002, *P*=0.009, *P*=0.068; Table 3). However, high YAP expressions with CEA (>5 ng/ml) play independent prognostic roles in both the single variable analysis and multivariate analysis (*P* < 0.001, *P*=0.011; Table 3).

**Figure 3:**
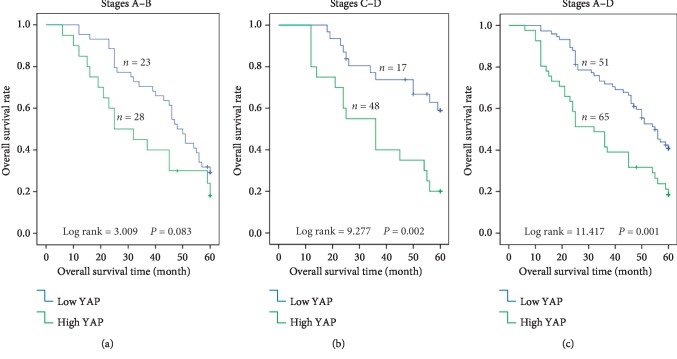
Relationship between YAP expression and overall survival of CRC patients. (a) For patients in the early clinical stage (stage A/B, *n* = 51), YAP protein levels in the cancer tissues were not clearly correlated with the overall survival rate (*P*=0.083). (b) For patients in the late clinical stage (stage C/D, *n* = 65), YAP protein levels in the cancer tissues were clearly related to poor overall survival (*P*=0.002). (c) For patients in all clinical stages (stages A–D, *n* = 116), YAP protein levels in the cancer tissues were clearly related to poor overall survival (*P*=0.001).

**Figure 4:**
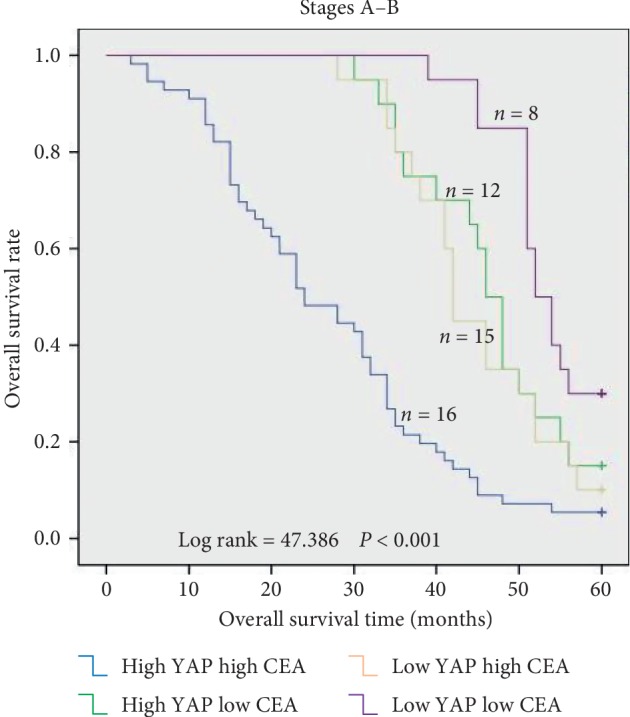
Relationship between YAP combined with CEA levels and overall survival of CRC patients in the early clinical stage (stage A/B, *n* = 51). For patients in the early clinical stage, high YAP levels in the cancer tissues and high plasma CEA levels were clearly related to poor overall survival (*P* < 0.001).

The relationship between YAP expression and the overall survival of CRC patients was evaluated using a Kaplan-Meier curve. For patients with Dukes stage A/B (*n* = 51), the YAP protein levels in the cancer tissues were not significantly related to the overall survival (*P*=0.083; Figure 3(a)). However, for patients with Dukes stage C/D (*n* = 65) and those with Dukes stage A–D (*n* = 116), the YAP protein levels in the tumor tissues clearly correlate with the overall survival (*P*=0.002, Figure 3(b); *P*=0.001, Figure 3(c)). The five-year survival rates of CRC patients with high YAP protein levels were clearly lower than those of CRC patients with low YAP protein levels. Furthermore, the correlation between YAP expression and overall survival was more significant in patients with the late clinical stage (stage C/D) (Figures 3(a)to 3(c)). These results reveal that YAP may be a significant prognostic factor for CRC patients in the late clinical stage, but not for those in the early clinical stage. We evaluated the relationship between YAP expression combined with CEA levels and the overall survival of CRC patients with Dukes stage A/B (*n* = 51). The high YAP protein levels in the cancer tissues combined with high plasma CEA levels (>5 ng/ml) were significantly related to the overall survival (*P* < 0.001; Figure 4). This indicates that YAP levels combined with plasma CEA levels are prognostic biomarkers for early-clinical-stage patients of CRC.

## 4. Discussion

To date, the upregulation of YAP has been implicated in tumor progression in different types of tumors such as pancreatic ductal adenocarcinoma, gastric cancer, NSCLC, and liver cancer [11–14]. Thus, YAP is characterized by the function of oncogenes. However, YAP acts as a tumor suppressor in breast cancer because of the deleted gene locus in the cancer cells, which results in low YAP expression [15]. Furthermore, some studies confirmed that YAP is a transcriptional coactivator that inhibits tumor growth through the interaction of YAP with p53-binding protein-2 and the p53 family member, p73 [16]. These results appear contradictory, suggesting that YAP is both a tumor suppressor gene and an oncogene. These results may be attributed to the dynamic changes in the Hippo-YAP cellular signaling pathways, which depend on the cancer type [17, 18]. Therefore, YAP may be playing a reversible role in different types of cancers. At present, the expression of YAP in CRC, its correlation with clinical pathologic features, and its prognostic value remain unclear.

In our study, the YAP expression levels in fresh CRC tissues and adjacent normal mucosae were measured via western blotting and qPCR. The results show that YAP protein and mRNA transcription levels in CRC tissues were clearly higher than those in the adjacent normal mucosae by 2 to 4 orders of magnitude. These results imply the correlation between high YAP expression and the progression of CRC tissues, which is consistent with other cancers in previous studies [11–14].

IHC results show that the positive signals for YAP were located mainly in the CRC cell nuclei. However, YAP was seldom detected in the matching adjacent normal mucosae. YAP expression levels in the CRC or lymphatic metastatic tissues were higher than those in normal mucosae. Furthermore, the YAP expression levels in the CRC tissues with lymphatic metastasis were clearly higher than those in the CRC tissues without lymphatic metastasis. These results suggest that YAP is involved in lymphatic metastasis. Moreover, YAP expression was found remarkably correlated with many clinicopathologic features such as serosal invasion, lymphatic metastasis, LNR, remote metastasis, and Dukes stage. The results show that YAP may be controlling the progression and metastasis of CRC. Furthermore, the close correlation between CEA and YAP indicates that YAP and CEA may be important markers for the diagnosis and prognosis of CRC. The Cox proportional-hazards model analysis confirms that YAP is a significant and independent prognostic marker for the overall survival rate of patients with CRC as well as for YAP combined with CEA. Moreover, Kaplan-Meier survival analysis results confirm that patients with high YAP expression had considerably lower five-year survival rates than those with low YAP expression. Further survival analysis reveals that YAP plays a significant role in the prognosis of late-stage CRC, thus indicating that YAP may be an important prognostic marker for late-stage patients. The five-year survival rates in CRC patients are consistent with those in NSCLC and HCC patients [13, 14]. Therefore, YAP is a significant prognostic factor whose upregulated expression is positively correlated with the poor overall survival rate in patients with CRC, particularly in those with the late clinical stage of the disease.

The biochemical gold standard for detecting CRC recurrence is CEA surveillance, and it is most effective when patients have high preoperative serum CEA levels [9]. However, there are some limitations. Sensitivity is far from being sufficient [19]. Plasma CEA level is not consistently elevated in CRC and may be undetectable or present at only low levels with poorly differentiated tumor [20]. There are efforts to improve the sensitivity and specificity using additional tumor markers like YAP. In this study, we found that high YAP levels in the cancer tissues and high plasma CEA levels were clearly related to poor overall survival in the early clinical stage. So YAP combined with plasma CEA may be appropriate prognostic biomarkers for early-clinical-stage patients of CRC.

However, our study shows that YAP itself has no significant relationship with overall survival in CRC early-stage patients. Because YAP expression level in early stage was lower than that in late stage. YAP level was positively related to Dukes stage and the CEA level. Currently, most studies consider YAP to be an oncogene that promotes cancer development [21, 22]. In contrast, some studies have reported that YAP can act as a tumor suppressor in various cancers [15, 23, 24]. However, it is impossible to simply categorize YAP as either a tumor promoter or a tumor suppressor. Nevertheless, it is widely accepted that dysfunction of Hippo signaling pathway cause abnormal accumulation of YAP within the cytoplasm and translocation of cytoplasmic YAP to the nucleus, where it functions as a transcriptional coactivator [25]. YAP cytoplasmic localization was correlated with inhibition of the Wnt signaling pathway, whereas YAP nuclear localization activated the Wnt signaling pathway, which played an essential role in colorectal carcinogenesis and CEA biosynthesis [23, 26]. We found that YAP was mainly present in the CRC cell nuclei in late stage, with small amounts YAP in the cytoplasm in early stage. These may explain why YAP itself has no significant relationship with overall survival in CRC early-stage patients. However, high YAP expression combined with high CEA level may reflect activation of oncogene and Wnt signaling pathway, which was positively associated with poor survival rates. The prognostic value of YAP combined with CEA in early-clinical-stage patients of CRC was observed in this study, although there are limitations of research on its mechanisms.

## 5. Conclusions

YAP is closely related to the progression and metastasis of CRC. Moreover, high YAP expression level is associated with poor overall survival of CRC patients, particularly in those with late clinical stage. High YAP levels in the cancer tissues combined with high plasma CEA levels are potential biomarkers for predicting CRC prognosis in the early clinical stage.

## Figures and Tables

**Table 1:** Relationship between YAP expressions and clinicopathologic features in colorectal cancers.

Features	Total	High YAP	Low YAP	*P*	*χ* ^2^
All case	116	76	40		
Age					
<56 years	55	37	18	0.706	0.143
≥56 years	61	39	22		
Gender					
Male	56	32	24	0.067	3.361
Female	60	44	16		
Tumor size					
<3 cm	45	31	14	0.543	0.370
≥3 cm	71	45	26		
Differentiation					
Low	15	10	5	0.712	0.137
Moderate	36	26	10		
High	65	40	25		
Serosal invasion					
N	46	25	21	0.040^*∗*^	4.209
Y	70	51	19		
Lymphatic metastasis					
N	43	22	21	0.013^*∗*^	6.232
Y	73	54	19		
LNR					
<28	54	30	24	0.035^*∗*^	4.438
≥28	62	46	16		
Remote metastasis					
N	90	54	36	0.020^*∗*^	5.410
Y	26	22	4		
Dukes stage					
A and B	51	28	23	0.033^*∗*^	4.540
C and D	65	48	17		
CEA					
≤5 ng/ml	40	20	20	0.011^*∗*^	6.507
>5 ng/ml	76	56	20		

*Note.* ^*∗*^Statistically significant (*P* < 0.05). Abbreviations: LNR, lymph node ratio. LNR = positive lymph node/total examined lymph node × 100%. Tumor size was measured by the length of the largest tumor nodule.

**Table 2:** Expressions of YAP in colorectal cancer tissues, metastasis lymphatic tissues, and adjacent normal mucosae.

Group	YAP expression				Total
	−	+	++	+++	
Adjacent normal mucosae	51	30	25	10	116^*∗*^^,#^
Colorectal cancer tissues	9	31	22	54	116^*∗*^
Lymphatic metastasis	7	12	12	42	73^&,$^
No lymphatic metastasis	2	19	10	12	43^&^
Lymphatic metastasis tissues	8	12	10	43	73^#,$^

*Note.* ^*∗*^Normal mucosae versus colorectal cancer tissues. *Z* = 16.037, *P* < 0.001, paired-sample *t*-test. ^#^Normal mucosae versus lymphatic metastasis tissues. *Z* = 9.322, *P* < 0.001, paired-sample *t*-test. ^&^Colorectal cancer tissues with lymphatic metastasis versus without lymphatic metastasis. *Z* = 4.875, *P*=0.017, independent-samples *t*-test. ^$^Colorectal cancer tissues with lymphatic metastasis versus lymphatic metastasis tissues. *Z* = −2.034 *P*=0.094, paired-sample *t*-test.

**Table 3:** Multivariate analyses of individual parameters for relationship with overall survival rate: Cox proportional-hazards model.

Variables	Single variable		*P*	Multivariable		*P*
	HR	95% CI		HR	95% CI	
YAP (high)	2.128	1.349–3.356	0.001^*∗*^	1.714	1.068–2.752	0.026^*∗*^
Age (≥56 years)	1.009	0.983–1.036	0.495			
Gender (female)	1.091	0.579–2.055	0.787			
Tumor size (≥3 cm)	1.014	0.940–1.095	0.713			
Differentiation (Y)	0.675	0.443–1.030	0.069			
Dukes stage (C and D)	2.187	1.427–3.350	<0.001^*∗*^	1.843	1.162–2.753	0.002^*∗*^
Lymph metastases (Y)	1.728	0.898–3.327	0.102			
LNR (≥28)	0.995	0.635–1.560	0.938			
Remote metastasis (Y)	4.247	1.802–10.009	0.001^*∗*^	3.131	1.333–7.355	0.009^*∗*^
CEA (>5 ng/ml)	1.667	1.054–2.637	0.029^*∗*^	1.535	0.969–2.433	0.068
YAP(high) + CEA (>5 ng/ml)	5.063	1.797–14.265	<0.001^*∗*^	3.936	1.367–11.331	0.011^*∗*^

Abbreviations: HR, hazard radio; CI, confidence interval; LNR, lymph node ratio. ^*∗*^Statistically significant (*P* < 0.05).

## Data Availability

The figure and table data used to support the findings of this study are included within the article.
